# Dual Interface Modification for Reduced Nonradiative Recombination in n–i–p Methylammonium-Free Perovskite Solar Cells

**DOI:** 10.1021/acsami.4c20462

**Published:** 2025-01-22

**Authors:** Juan José Rodriguez-Perez, Diego Esparza, Muhammad Ans, David Armando Contreras-Solorio, Teresa Diaz Perez, Jhonatan Rodriguez-Pereira, Eva M. Barea, Isaac Zarazua, Daniel Prochowicz, Seckin Akin, Juan P Martinez-Pastor, Jorge Pascual, Iván Mora-Seró, Silver-Hamill Turren-Cruz

**Affiliations:** † Unidad Académica de Ciencia y Tecnología de la Luz y la Materia, 27779 Universidad Autónoma de Zacatecas, Carr. Zacatecas-Guadalajara km 6, Ejido La Escondida, 98160 Zacatecas, Mexico; ‡ Institute of Advanced Materials (INAM), 16748 Universitat Jaume I, Av. Vicent Sos Baynat, s/n, 12071 Castellón de la Plana, Spain; § Unidad Académica de Ingeniería Eléctrica, Universidad Autónoma de Zacatecas, Av. Ramón López Velarde 801, Col. Centro, 98060 Zacatecas, Mexico; ∥ Department of Physical Chemistry, 119463 Polish Academy of Sciences, Warsaw 01-224, Poland; ⊥ Center of Materials and Nanotechnologies, Faculty of Chemical Technology, 48252 University of Pardubice, Nam. Cs. Legii 565, 53002 Pardubice, Czech Republic; # Central European Institute of Technology, Brno University of Technology, Purkyňova 123, 612 00 Brno, Czech Republic; ∇ Centro Universitario de los Lagos, 42561 Universidad de Guadalajara, Lagos de Moreno 47460, Jalisco, Mexico; ○ Department of Metallurgical and Materials Engineering, 226846 Necmettin Erbakan University, 42060 Konya, Turkey; ◆ Instituto de Ciencia de los Materiales (ICMUV), 253325 Universitat de Valencia, 46980 Paterna, Spain; ¶ Polymat, University of the Basque Country UPV/EHU, 20018 Donostia-San Sebastian, Spain

**Keywords:** interface modification, 2D/3D perovskite, perovskite solar cells, perovskite stability, halide segregation

## Abstract

High defect concentrations at the interfaces are the basis of charge extraction losses and instability in perovskite solar cells. Surface engineering with organic cations is a common practice to solve this issue. However, the full implications of the counteranions of these cations for device functioning are often neglected. In this work, we used 4-fluorophenethylammonium cation with varying halide counteranions for the modification of both interfaces in methylammonium-free Pb-based n–i–p devices, observing significant differences among iodide, bromide, and chloride. The cation treatment of the buried and top interfaces resulted in improved surface quality of the perovskite films and largely improved carrier dynamics with reduced nonradiative recombination. Consequently, the optimal interface-modified methylammonium-free perovskite solar cells surpassed 20% efficiency and demonstrated remarkable operational stability. Our findings underscore the potential of comprehensive surface engineering strategies in advancing the perovskite film and device quality, thereby facilitating their broader and more successful applications.

## Graphical abstract

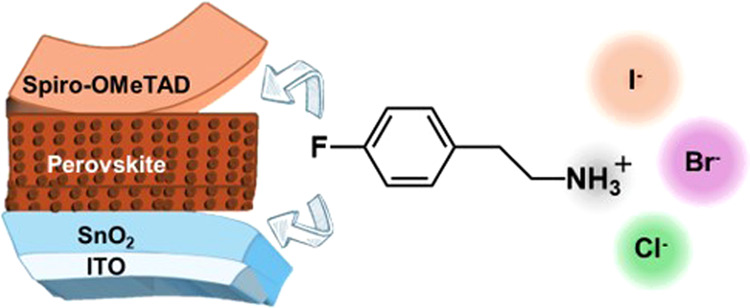

## Introduction

In recent years, perovskite materials have gained significant interest due to their optoelectronic properties and low production cost.^1,2^ Among other benefits, they present an excellent absorption coefficient, a broad absorption spectrum, and an adjustable band gap.^3,4^ This has led to rapid improvements in the efficiency of perovskite solar cells (PSCs) over the past decade, with record efficiencies now exceeding 26%.^5,6^ The quick development of PSCs has been in part due to the implementation of interface engineering strategies, which are gaining increasing popularity in the field.^7–9^ Interfaces are critical components of optoelectronic device functioning in terms of defect chemistry and charge dynamics.^10,11^ In addition, they have a key role in material and device stability, as the high concentrations of defects at these surfaces are a source of degradation, leading to increased nonradiative recombination losses and decreased voltage.^12,13^ Therefore, interface modification strategies open the door to a reduction of the defect density and a more favorable electronic configuration (e.g., energy level alignment). Optimized surfaces enhance charge extraction and reduce charge recombination, ultimately improving the device’s efficiency and stability.

Furthermore, the buried interface also plays a critical role in determining the crystallization of the perovskite thin film. This aspect has gained more attention from the perovskite community in recent years, with self-assembled monolayers as the most notable example.^14^ Common interface modifications, both at the bottom or on top of the perovskite layer, generally involve defect passivation with the introduction of inert polymers,^15^ or organic cations like phenethylammonium,^16^ butylammonium,^16,17^ or ethylenediammonium,^15,18,19^ or controlling the dimensionality of the surface according to the chemical behavior of the employed modifier.^20–22^ Despite the effectiveness of these strategies in enhancing device efficiency, correctly evaluating the inertness of these modifications and their compatibility with perovskite remains a critical aspect in order to rule out the introduction of any possible additional decomposition route.

In this work, we present the dual interface modification of Pb-based MA-free PSCs with an n–i–p architecture with a functional organic salt containing different counteranions. We particularly investigated the influence of 4-fluoro-phenethylammonium (4FPEA-X) modifier with different halide counteranion (Cl^–^, Br^–^, I^–^) on both top (perovskite/hole-transporting layer (HTL)) and buried interfaces (electron-transporting layer (ETL)/perovskite). While 4FPEA-X and similar compounds are widespread organic salts for interface modification,^23–25^ reported works do not deepen on the importance of the surfactant counteranion nature toward device efficiency and stability. The results indicate that the surface modification with any of the studied counteranions effectively passivates defects at the perovskite surfaces, which reduces the nonradiative recombination losses and leads to a general increase in the performance of the solar cells to up to 20%. However, the selection of the counteranion is crucial to guarantee high stability in the devices. In particular, the introduction of Br^–^ or Cl^–^ results in a severe reduction of the long-term stability of PSCs, losing over 20% of the initial efficiency in a matter of a few hours, in contrast to devices with I^–^-based modifiers that remained largely stable.

## Experimental Section

### Reagents

The SnO_2_ colloid precursor was purchased from Alfa Aesar (tin­(IV) oxide, 15% in H_2_O Colloidal dispersion). Cesium iodide (CsI, 99.99%), 2,2′,7,7′-tetrakis­[*N*,*N*-di­(4-methoxyphenyl)­amino]-9,9′-spirobifluorene (spiro-OMeTAD, 99.86%), and lead­(II) iodide (PbI_2_, 99.99%) were purchased from TCI. Formamidinium iodide (FAI), 4-fluoro-phenethylammonium iodide (4-FPEAI), bromide (4-FPEABr), chloride (4-FPEACl), and dipropylammonum iodide (DipI) were purchased from Greatcell Solar Materials. Dimethyl sulfoxide (DMSO, anhydrous, >99.9%), *N*,*N*-dimethylformamide (DMF, anhydrous, 99.8%), chlorobenzene (CB, anhydrous, 99.8%), hydroiodic acid (stabilized), 4-*tert*-butylpyridine (tBP), bis­(trifluoromethylsulfonyl)­imide lithium salt (LiTFSI), ethanol, 2-propanol (IPA), and acetonitrile were purchased from Sigma-Aldrich. All of the reagents were used without further purification.

### Solar Cell Fabrication

ITO-coated glass substrates are ultrasonically washed for 15 min with soap, deionized water (Milli-Q H_2_O), acetone, and ethanol. The colloidal solution for the compact layer of SnO_2_ was prepared by mixing in a vial with1.0 mL of 15% SnO_2_ (precursor) in 1.0 mL of Milli-Q water. Before spin coating, ITO-coated glass substrates are treated with ultraviolet ozone (UVO) for 20 min. Then, 120 μL of solution of SnO_2_ was added and spin-coated at a speed of 4000 rpm and acceleration of 1000 rpm/s for 40 s, and every sample was annealed at 150 °C for 30 min.

The bottom interface was modified with 4-FPEAI by processing a 0.02 M solution in DMF on the SnO_**2**_ substrates by spin coating at 4000 rpm for 40 s. The 1.4 M perovskite precursor solution was prepared by dissolving 213.97 mg FAI, 8 mg DipI (for *n* = 80), 35.95 mg CsI, and 645.4 mg PbI_2_ in the mixture in 1 mL of DMF:DMSO (4:1) solvent mix and heating it at 66 °C for 4 h before deposition. The perovskite films were deposited by dripping 80 μL of perovskite precursor solution on the SnO_2_ passivated films and spin coating at 6000 rpm for 40 s, with 100 μL of CB antisolvent dripped onto the perovskite films 25 s after the program started. The films were then annealed at 150 °C for 10 min. The top interface was modified with 4-FPEAI at a concentration of 2.3 mg/mL, 4-FPEABr at 2 mg/mL, and 4-FPEACl at 3 mg/mL in IPA, spin-coated at 4000 rpm for 40 s. The HTL was prepared by dissolving 72.3 mg of spiro-OMeTAD, 28.8 μL of TBP, and 17.5 μL of LiTFSI (from a solution of 520 mg of LiTSFI in 1 mL of acetonitrile) in 1 mL of CB and dynamically depositing 50 μL after 2 s by spin coating at 4000 rpm and 800 acceleration for 40 s. Finally, 80 nm thick Au electrodes are thermally evaporated onto spiro-OMeTAD film under a 1 × 10^–6^ mBar vacuum using a shadow mask.

### Characterization

Scanning electron microscopy (SEM) characterization was carried out with a field emission scanning electron microscope (FEGSEM JEOL 3100F) operated at 15 kV. XRD pattern of the films was measured using an X-ray diffractometer (D8 Advance, Bruker-AXS) (Cu Kα, wavelength λ = 1.5406 Å) with a Bragg angle range of 5° to 50° and step size of 0.05°. The perovskite film absorption spectra were registered on a Varian 20Cary300BIO UV/vis spectrophotometer. The steady-state PL measurements were registered by using an intensified CCD (iStar 320 ICCD/DH320T-25U-03) coupled to a spectrograph (KYMERA-328I-B1) from Andor. A continuous diode-pumped solid-state laser module (532 nm, 150 mW max) with controllable output power, model GL532RM-150 from SLOC LASERS, was used as an excitation source. Time-resolved photoluminescence (TRPL) of thin films was measured with an Edinburgh Instruments model FLS1000 spectrophotometer under continuous excitation with a Xenon (Xe2) lamp. The detector is a single photon counting PMT with a signal noise ratio of 20,000:1. Atomic force microscopy (AFM) (Concept Scientific Instrument) was performed in Kevin probe force microscopy (KPFM). The images (10 × 10 μm^2^ in size) were recorded using a Pt-coated tip in a noncontact variant. The roughness average (*R*_a_) was measured on topography images at 5 μm on the ordinate axis. Ultraviolet photoelectron spectroscopy (UPS) measurements were performed by a PHI 5000 VersaProbe with a He I source (21.22 eV) under an applied bias of 7.0 V. Photocurrent voltage (*J*–*V*) curves were measured using an Abet Technologies solar simulator in room atmosphere conditions. The light intensity was adjusted to 1 sun (100 mW/cm^2^). The devices were measured with an active area of 0.121 cm^2^, defined by a mask. The stabilized power output was acquired using an MPP tracking algorithm for 500 s. Devices were not preconditioned before measurement. Masking was used during the measurement, defining a pixel area of 0.10 cm^2^. Aging tests were also performed using a Fluxim Litos setup and 1 sun equivalent illumination with no UV component, holding the substrates at 45 ± 5 °C in an N_2_ atmosphere and using an MPP tracking algorithm. Maximum power point tracking (MPPT) and long-term stability measurements in room atmospheric conditions were measured under continuous illumination, using a xenon lamp, by adjusting the sample-light source distance to set an optical power density of 100 mW cm^–2^, calibrated with a photodiode. The measurements were performed with an AUTOLAB (PGSTAT30) potentiostat; for the long-term stability measurements, an automatic sequence was set to measure the *J*–*V* curves every 2h. For the MPPT measurements, a fixed voltage corresponding to *V*_MPP_ was applied, which was obtained from the *J*–*V* curves, and the electrical current *I*_MPP_ was monitored over time. V_MPP_ is directly extracted from the *J*–*V* curves. It is important to highlight that MPP stability measurements were not performed using an MPP tracker but at a fixed voltage, and consequently, strictly speaking, our experiment could be considered a quasi-MPP experiment. External quantum efficiency (EQE) measurements were performed with a QEPVSI-b Oriel system. The surface chemical composition and electronic state of perovskite films-4F were analyzed by X-ray photoelectron spectroscopy (XPS, ESCA-2SR, Scienta-Omicron). Spectra were recorded using a monochromatic Al Kα = 1486.6 eV X-ray source operated at 200W. The binding energy scale was referenced to adventitious carbon (284.8 eV). CasaXPS software (Casa Software Ltd.) was used to analyze the data, and the quantitative analysis was made using sensitivity factors provided by the manufacturer. Electrochemical impedance spectroscopy (EIS) was measured using a Potentiostat Autolab PGSTAT30, employing different filters to change light intensity up to 1 sun. For each voltage point (*V*_OC_), EIS was measured with an AC 10 mV voltage perturbation from 1 mV to 100 MHz. Nova software was used to generate data, and Z-View software was used to model the equivalent circuit model used to fit the spectra.

## Results and Discussion

We fabricated full devices on a planar stack of glass/ITO/SnO_2_/4FPEA-X/perovskite/4FPEA-Y/spiro-OMeTAD/Au (where ITO stands for indium tin oxide) depicted in Figure S1; see further fabrication details in the Supporting Information. We used MA-free perovskite films with Cs_0.1_FA_0.9_PbI_3_ composition, where FA stands for formamidinium. In successive steps, we have optimized the fabrication conditions. We introduced dipropylammonium iodide (DipI), where Dip^+^ is a bulky organic cation additive with the ability to improve the performance and stability of PSCs.^17,25,26^ See Figure S2 for a detailed optimization of the Dip^+^ concentration in the film. In Figure S3, we first used 4FPEA-I as a modifier of the bottom interface and prepared solar cells with the above-mentioned structure to find the optimum concentration at which to compare the different interlayers. In Figure S4, we compare 4FPEA-X bottom interface modifiers with the different halides at the previously optimized concentration, finding that the Br-based modifier leads to the highest average performance, but the I-based one presents higher dispersion, including also the singular devices with the highest photoconversion efficiency (PCE). Overall, all modifiers led to very reproducible results. Therefore, looking for the champion performance, we set 4FPEA-I as the bottom interface modifier that we will use for studying the top interface modification. In order to find the best concentration at which to apply the modifiers at the top interface, in Figure S5, we applied 4FPEA-I at different concentrations, where we observed that the best condition is 1 mg/mL. Thus, in the present study, we compared the application of the different interlayers at a concentration of 1 mg/mL in solar cell structures, where we modified the bottom interface with 4FPEA-I.

Here, we used 4FPEA-X (I^–^, Br^–^ or Cl^–^) interface modifiers in order to understand the impact of the halide counteranion component in the same perovskite and device structure system. For brevity, we denoted these compositions as 4F-X (where X: I^–^, Br^–^ or Cl^–^). For the case of the dual interlayer modification, we denoted the samples as 4F-X/Y; X is related to the halogen used at the bottom interface (ETL/perovskite), and Y is related to the halogen used at the top interface (perovskite/HTM).

We examined the influence of surface modification by 4F-X and its halogen nature on the perovskite film morphology by scanning electron microscopy (SEM). In Figure 1a, we noticed that the samples treated with interface modification agents exhibit small grains on the surface atop the perovskite films. We ascribe these bright formations to PbI_2_, according to our previous findings in double and triple-halide perovskite films,^27^ probably due to interface reconstruction by the organic modifier. We also observed that samples 4F–I/I and 4F–I/Cl show a slight enhancement in surface flatness over the 4F–I/Br and control samples. All of the samples presented comparable average grain sizes between 520 and 570 nm, as shown in Figure S6 and Table S1. However, we observed that the difference is insignificant; all samples represent grain sizes of between 1100 and 1340 nm.

**Figure 1:**
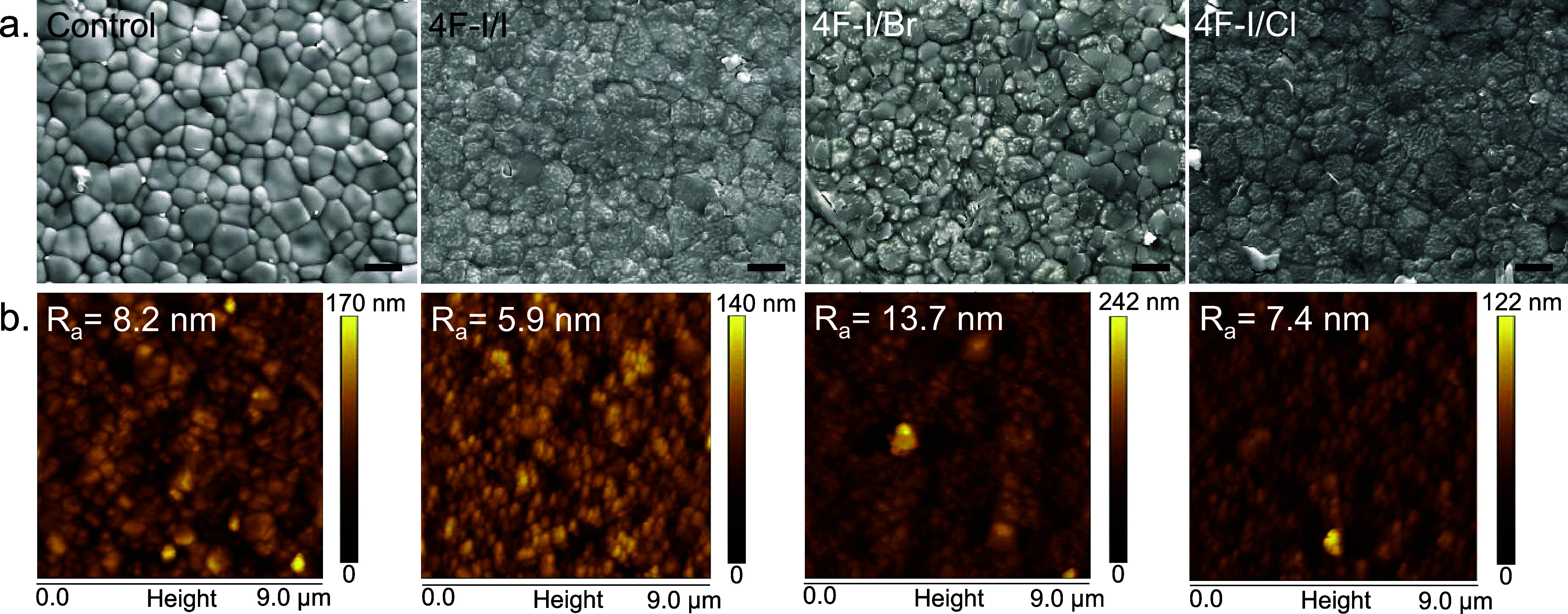
(a) SEM and (b) AFM images for control and interface-modified perovskite thin films. The scale bar is 1 μm.

To better understand the influence of interface modification on the morphology of the samples, we analyzed their surfaces by atomic force microscopy (AFM). Figures 1b and S7 show how the surface morphology is much more homogeneous and compact, with a very homogeneous height distribution after the interface modification. For the particular case of the 4F–I/Cl sample, there is a slight increase in the grain size, and the surface of the sample is homogeneous. We found a comparable homogeneous surface for sample 4F–I/I. Thus, both surface-modified structures have great potential for high conversion efficiency in solar cells. On the contrary, the interface modification with 4F–I/Br was not as effective, showing a less homogeneous surface with many high-potential points that can harm the final efficiency of the device.

To further investigate the influence of the top interface modification on the crystalline properties of the perovskite films, we analyzed the X-ray diffractograms for the control and 4F–I/I, 4F–I/Br, and 4F–I/Cl samples; see Figure 2a. The corresponding surfactant salt (4F-X) structure is present in all samples, as evidenced by diffraction peaks at 5.4 and Dip-based 2D structure at 7.9°.^17^ The sample 4F–I/Br presents a nonperovskite signal for the δ-FAPbI_3_ phase at 10.78°, which is linked to material degradation in PSCs.^15,28^ While iodide replacement with bromide can stabilize the α-FAPbI_3_ phase, bromide in concentrated amounts has been reported to also induce δ-FAPbI_3_ generation.^29^ This could be caused by the penetration of bromide into the perovskite film and its incorporation in the lattice, restructuring it partially and promoting a reversible formation of perovskites with different halide content. This could in turn end up in the segregation of pure FAPbI_3_ material that may tend to form in the δ phase. Furthermore, this XRD presents a broad peak at 14.0°, previously ascribed to horizontal (111) preferential growth in Dip-based 2D perovskites and the (101) plane in 3D perovskites.^30^ The characteristic peaks of 3D perovskites at (101), (110), (202), (222), (400), and (330) are present in the samples.

**Figure 2:**
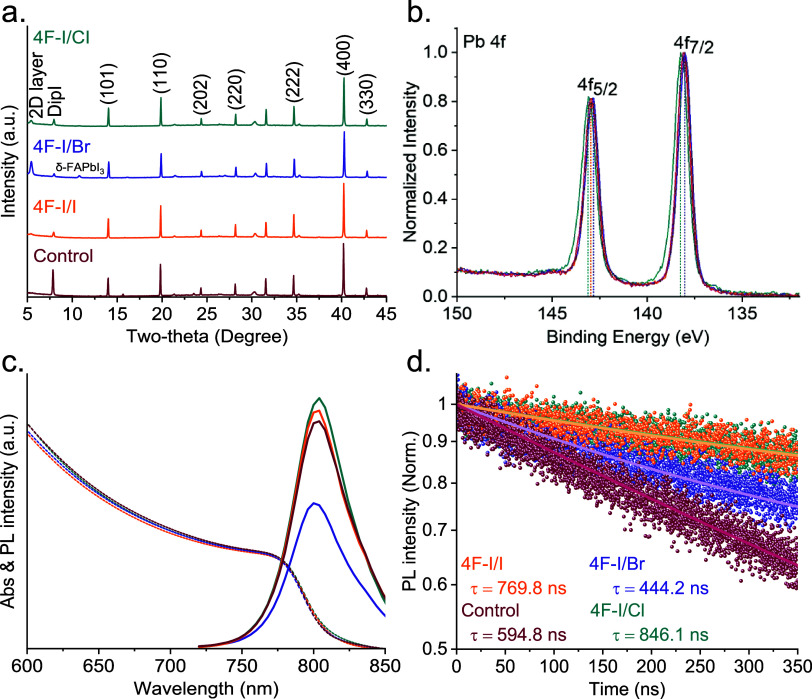
(a) XRD patterns, (b) XPS spectra of Pb 4f, (c) PL and UV–vis absorption spectra, and (d) TRPL-decay for perovskite films with and without interface modifications.

To investigate the elemental distribution on the surface of different perovskite films, we performed X-ray photoelectron spectroscopy (XPS) measurements on the four samples; see Figures 2b and S8. In the case of control films, we can identify the FA components by specific peaks in the C 1s spectra at 288.1 and 284.9 eV, see Figure S8a, and in the N 1s spectra at 400.3 eV, see Figure S8b. As XPS is a superficial technique and thus probes a few nanometers of the top section, we could identify the presence of the different 4F-X salts applied as surface modifiers. We could distinguish these components by a specific peak at a binding energy of 286.5 eV in the C 1*s* spectra originating from the carbon backbone in the surfactant structures, see Figure S8a. Moreover, the single peaks located in the N and F 1s spectra at 401.7 and 687.1 eV, respectively, belong to the C–N bonds in the amino groups and the characteristic fluoro-substitution on the phenyl groups, see Figure S8b,c. For the Pb 4*f* spectra in the CsFAPbI_3_ perovskite structure, two Pb 4f_7/2_ and Pb 4f_5/2_ peaks are observed at 138.3 and 143.2 eV, respectively, see Figure 2b. In the case of sample 4F–I/Cl perovskite, both Pb 4f_7/2_ and Pb 4f_5/2_ shift 0.1 eV toward higher binding energy, in comparison with 4F–I/I that does not present any shift and 4F–I/Br that decreases 0.1 eV. This result suggests a potential influence of chloride in the environment of Pb, such as the passivation of superficial undercoordinated Pb^2+^ cations. Meanwhile, the Cs and I 3*d* and O 1*s* spectra did not show any significant difference among samples, see Figure S8e,f.^31^ Overall, XPS reveals the effective deposition of 4F–I/Y salts on top of the perovskite surface and anticipates a potential benefit of Cl-based salts through Pb-based defect passivation. The surface compositions of the different samples are summarized in Table S2.

We performed ultraviolet photoelectron spectroscopy (UPS) measurements in order to study the energy alignment of the perovskite film treated with the different materials tested (Figures S9 and S10), with direct implications for their photovoltaic performance. Although all surface modifiers raise the valence band maximum (VBM) of the perovskite film, the modification with 4F–I/I raises it the most (5.65 eV), improving its alignment with the HOMO of the hole-selective layer and indicating efficient hole extraction with minimal energy loss, potentially contributing to superior photovoltaic efficiency. Meanwhile, 4F–I/Br and 4F–I/Cl caused a lower increase of the VBM (−5.72 and −5.74 eV, respectively), showing a poorer alignment and thus making them less ideal candidates for enhancing photovoltaic performance in the solar cells compared to 4F–I/I. Overall, this analysis suggests that 4F–I/I, with its optimum energy alignment, offers the best trade-off for charge extraction efficiency and minimal recombination losses, making it the most promising candidate for high-efficiency photovoltaic devices among the materials analyzed.

We measured all samples’ absorption spectra and photoluminescence to study the effect of the different surface modifications on the perovskite band gap. All samples present the absorption edge around 850 nm (E_g_ = 1.54), indicating that the introduction of these interface modifiers does not change the band gap of the perovskite film, see Figures 2c and S11, and Figure S12 for the energy level diagram of the perovskite film with the different interface modifications. Comparing the PL intensity, we can see that 4F–I/I and 4F–I/Cl have more intense peaks than the control sample, while 4F–I/Br has a significant PL. The PL intensity increase in these samples suggests a reduction of the nonradiative recombination, pointing to a passivation of defects on the surface or within the grains of the perovskite films. PL peaks are located at about 780 nm, which is consistent with previous reports in the literature.^32^ We performed time-resolved photoluminescence (TRPL) measurements to investigate the recombination mechanisms. The obtained lifetime values are shown in Table S3. For the control sample, an effective lifetime of 594.8 ns was obtained, while for the 4F–I/Cl, 4F–I/Br, and 4F–I/I samples, the values were 846.1, 444.2, and 769.8 ns, respectively. The fast component can be assigned to the rapid carriers recombination across the perovskite and electron-transporting layer interfaces.^33^ Thus, the 4F–I/Cl and 4F–I/I-based perovskite films showing the highest τ_ef_ and τ_f_ present the reduced carrier recombination at the interface, compared to the control and 4F–I/Br samples.

Figure 3a shows a full n–i–p device structure and cross-sectional SEM for the 4F–I/I sample, where the interlayers cannot be observed due to their too-small thickness (not visible by SEM); the scale bar is 200 nm. Devices modified with 4F–I/Y showed significantly improved efficiencies. The top-performing control device provided an efficiency of 17.83%, which was greatly improved for all interface-modified champion devices, i.e., 4F–I/Cl (19.28%), 4F–I/Br (19.27%), and particularly 4F–I/I for the record value of 20.00%, see Figure 3b and Table S4 for summarized photovoltaic parameters. The short-circuit current (*J*_SC_) was 22.40 mA cm^–2^ for control, 22.50 mA cm^–2^ for 4F–I/Br, 22.64 mA cm^–2^ for 4F–I/I, and 22.41 mA cm^–2^ for 4F–I/Cl, which are in good agreement with those values integrated from the external quantum efficiency (EQE) spectra of 21.50, 21.78, 22.33, and 22.08 mA cm^–2^, respectively, see Figure 3c. The EQE spectra show a photoresponse from the UV (≈400 nm) to the infrared (≈800 nm) region, where a small increase in the photocurrent response is observed for the samples with interlayer modification, denoting a higher efficiency in the conversion of photons to electrons in the devices. The champion device based on the 4F–I/I interface modification with 20.0% power conversion efficiency (PCE) exhibited an open-circuit voltage (*V*_OC_) of 1.11 V and a fill factor (FF) of 78%. In Figure S13, we see that devices with a 4F–I/Y interlayer show some of the highest PCE and exhibit more reproducible device parameters, especially in terms of *V*_OC_ and FF, highlighting the potential of interfacial engineering. According to the PL results, we would expect a better performance for control devices in comparison to 4F–Br-containing ones. However, both FF and *V*_OC_ increase for the latter. Therefore, we speculate that introducing any surface modification, even a not-ideal one, has the potential to outperform a nonmodified device, at least in terms of PCE.

**Figure 3:**
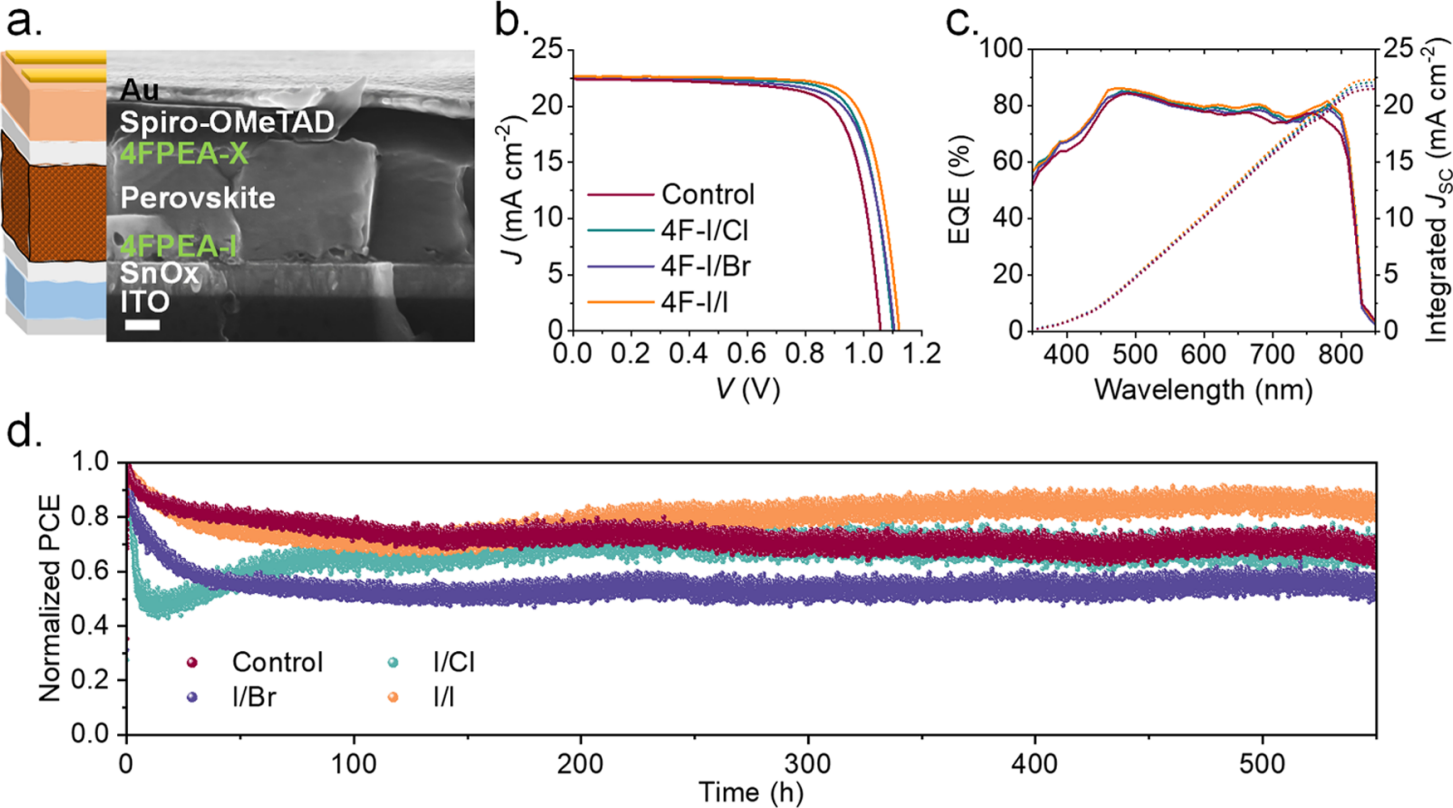
(a) Full n–i–p device structure and cross-sectional SEM for the 4F–I/I sample. The scale bar is 200 nm. (b) *J*–*V* curves of the best-performing control and interface-modified devices, (c) the corresponding EQE spectra, and (d) MPPT measurements at 1 sun illumination without encapsulation in N_2_ atmosphere at 25 °C.

The samples 4F–I/Cl, 4F–I/Br, and 4F–I/I exhibit an enhancement in *V*_OC_, with higher average values of 1.09, 1.07, and 1.10 V, respectively, compared to the 1.05 V of the control sample. In a similar way, FF shows an increase for the interface-modified samples with 75, 76, and 77% compared with 72% of the control sample, as observed in Table S4. The average PCE presents an 18.3, 17.8, and 18.7% enhancement for 4F–I/Cl, 4F–I/Br, and 4F–I/I, respectively, compared to the control 16.5%. On average, the devices present comparable *J*_SC_, with just a slight increase observed for the 4F–I/I and 4F–I/Cl samples; see the example of the behavior of champion devices in Figure 3b. Thus, the increase in PCE originates predominantly from *V*_OC_ and FF. These results are in line with previous observations on the improved morphological and optoelectronic properties of the interface-modified perovskite films, where these interlayers may be playing a crucial role in passivating surface defects, diminishing recombination, alleviating the adverse effects of pinholes, and preventing the migration of metal electrodes at elevated temperatures, ultimately leading to significant improvements in charge extraction at the interfaces and enhancing *V*_OC_ and FF.^34^

In addition to solar cell efficiency, material and device stability is the main current challenge for PSC commercialization. To analyze the impact of our interface modification strategy on device stability, we tracked devices at the maximum power point (MPP) for 550 h at 1 sun illumination in an N_2_ atmosphere at 25 °C, see Figure 3d. We observe that the control device shows a notable loss in performance of about 33% after over 500 h. The 4F–I/Br and 4F–I/Cl devices decay by 26 and 24% from their initial efficiency. Although the sample 4F–I/Br and 4F–I/Cl improve the *V*_OC_ and FF compared to the control sample, these interface modifications induce a much faster degradation of the solar cells than the control sample, particularly with a fast decay during the 1st hours of tracking. We ascribe the recovery present in the 4F–I/Cl sample after the decline of the 1st hours to the impact of the Dip additive, according to previous observations showing the same behavior.^24,26^ Meanwhile, the 4F–I/I device shows a significant improvement in stability compared with the other samples, with an efficiency loss of only around 17%. In the same way, long-term stability was measured for 80 h under continuous illumination (100 mW cm^–2^, AM1.5G) at a constantly applied voltage, without encapsulation at 70% RH without temperature control (temperature reaching 60 °C due to lamp heat radiation), see Figure S14. The sample 4F–I/Br degrades completely within a few minutes of its measurement, 4F–I/Cl degrades exponentially within 15 h under illumination, and the control sample shows quasi-linear degradation within 45 h of illumination. Finally, the sample containing only 4F–I/I proves to be the most stable, as it presents a linear degradation in the first 10 h, slightly better than the control sample, and from that time on, it presents a significantly more moderate logarithmic degradation than the control sample, to up to 83 h under light.

One of the main problems of interface engineering is, in fact, that they increase the efficiency of the devices but, in certain cases, originate from a detriment of stability. Designing interface modification strategies that increase device performance without sacrificing device robustness is in fact one of the main challenges of this type of strategy. Our study observed that stability worsens for chloride and bromide but is maintained for iodide. Thus, our findings indicate that surface modifications introducing non-native anions such as Cl^–^ and Br^–^ can induce degradation linked to halide segregation.^27,35^ Thus, we present here a highly relevant aspect to consider for surface engineering strategies, pointing out that the proper selection of concentrations in organic salts can avoid the introduction of additional degradation routes at the interfaces. Employing smaller anions such as chloride favors the coordination of metal centers (e.g., Pb^2+^, Sn^2+^), particularly the undercoordinated ones. It will, therefore, be positive for the passivation of defects but at the expense of introducing new decomposition pathways. Furthermore, these findings are highly relevant for understanding the impact on the stability of interface modification strategies in mixed halide perovskite systems. In this study, we studied the influence of halides on iodide-pure perovskites. In this sense, the composition of the perovskite film may be as important as the composition of the surface modifier, taking into account that the halide segregation introduced by the organic salt is detrimental, particularly due to the iodide purity of the absorber film. In mixed halide systems, which have widespread use and their stability is of utter importance for field advancement, organic salts may present different working mechanisms, potentially introducing new degradation pathways, accelerating present ones, or even preventing them, making this an aspect worth exploring in the field. Similarly, we anticipate this same phenomenon for any type of organic salt with a specific halide, e.g., the common phenethylammonium (PEAX) or butylammonium (BAX), independently of its structure.

We performed impedance measurements under open-circuit conditions and different irradiances to better understand how the interface modifiers affect the recombination processes (which are closely related to the *V*_OC_ behavior) of the fabricated solar cells. Figure S15a shows an example of the typical Nyquist plots obtained for all of the analyzed samples, which show two well-defined arcs. To properly analyze the impedance response, the spectra were fitted using the circuit shown in Figure S15b,^36^ where each component of the circuit was associated with different electric and ionic processes inside the cells.^35,37^ The sample with 4F–I/Br is excluded from the impedance analysis since it degraded quickly, and it was impossible to obtain reliable results, as shown in Figure S14. Then, we plotted the obtained circuit component values against the measured *V*_OC_, as shown in Figures 4 and S16. Analyzing in detail the series resistance, *R*_s_, see Figure 4a, gives us information about the quality of the contacts; we found that the control sample has an *R*_s_ of approximately 1.5 Ω·cm^2^, i.e., a promising value for a solar cell. The samples 4F–I/Cl and 4F–I/I have lower *R*_s_, indicating a softer interface in these materials, the best being 4F–I/I with less than 1 Ω·cm^2^. These results suggest that the higher FF observed in the samples with interface modification layers is partially due to a better electrical contact promoted by the 2D materials, which is in line with AFM analysis.

**Figure 4:**
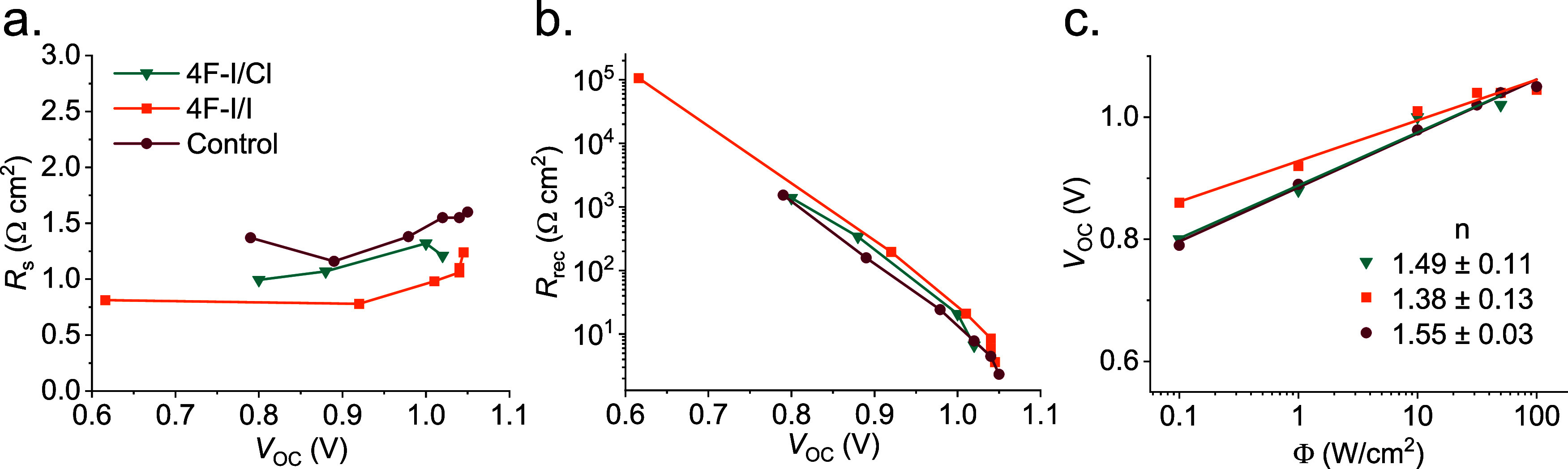
Impedance spectroscopy of the control and interface modification. (a) Series resistance, (b) recombination resistance vs *V*_OC_, and (c) ideality factor “*n*” calculation from the *V*_OC_ vs irradiance dependence.

When analyzing the recombination resistance, *R*_rec_, see Figure 4b, we found that the control sample presents the typical exponential behavior, going from 1000 to 1 Ω cm^2^ between 0.8 and 1.2 V.^36–38^ The modified contacts are obtained by placing parallel lines that run above the control sample. This means that the samples 4F–I/Cl and 4F–I/I inhibit the recombination processes, as reflected in the increase of *R*_rec_, being in ascending order of inhibition 4F–I/Cl < 4F–I/I. It is important to highlight that *V*_OC_ is strongly influenced by the recombination process, i.e., proportional to the inverse of *R*_rec_, following the same increase trends, see Figures 3 and S13. It could be concluded that the increase in *V*_OC_ is principally due to the reduction in the interfacial recombination processes given by the passivation layers. The *V*_OC_ and FF are correlated with nonradiative recombination processes in PSCs,^39^ thus optimal *V*_OC_ and FF are obtained by reducing the density of defects, thus increasing carrier lifetime and suppressing nonradiative recombination.^39,40^

As a parallel analysis, we can plot the *V*_OC_ against the irradiance to measure the ideality factor n, see Figure 4c, of the different samples.^41^ We found that the control sample has an n of 1.55, suggesting a hybrid recombination process between surface and nonradiative recombination (Shockley–Read–Hall, SRH, and surface recombination).^38–42^ The sample 4F–I/Cl showed an n value similar to the control sample, while for the 4F–I/I sample, *n* decreased to 1.3, which, together with the increase of *V*_OC_, suggests a reduction in nonradiative recombination processes, which could also be partially responsible for the higher FF these samples, see Table S4. However, it must be considered that the margin of error in the regression indicates that the control, 4F–I/Cl, and 4F–I/I are statistically equal, as can be seen in Figure 4c. Finally, the ion-related resistance and capacitance, *R*_ion_ and *C*_ion_, respectively, give information about the ionic behavior of the samples.^36–43^ Its analysis must be simultaneous; both the behavior of the resistance and that of the capacitance must be seen. 4F–I/Cl and 4F–I/I samples have higher *R*_dr_ values than the control sample and, at the same time, have lower *C*_dr_. This behavior points to a decrease in the vacancy density due to the interfacial modification.^43^

## Conclusions

Perovskite solar cells often present imperfect interfaces with inefficient charge extraction. Moreover, interface modification strategies tend, in some cases, to introduce further decomposition pathways, sacrificing device stability for a slight increase in efficiency. In this work, we present the careful optimization of the bottom and top interfaces in n–i–p MA-free PSCs with 4FPEA-X surface modifiers based on different halides (X: Cl, Br, I). The introduction of a 4FPEA-I interlayer at the bottom interface led to the best champion photoconversion performance in devices with respect to the other modifiers. Meanwhile, the top interface modification led to significant differences in the morphological and optoelectronic properties depending on the halide content. Modifiers based on chloride and iodide led to the best perovskite surfaces, which translated into increased FF and *V*_OC_. Overall, our results indicate that the surface modification of n–i–p MA-free perovskites with any of the studied counteranions passivate surface defects and reduce the recombination processes, increasing the PSC performance to 20%. However, the selection of the counteranion is crucial to guarantee high-stability devices. Interface engineering strategies, in some cases, reduce device stability by introducing further decomposition mechanisms. In this work, we find that using organic surfactants based on iodide for the modification of perovskites composed solely of this same anion can avoid this additional degradation route, as observed when incorporating 4FPEA-I. The interface modification with organic material is a strategy with a lot of potential, as it increases efficiency, but all of the factors must be closely controlled, including the counteranion, since it can negatively affect the stability. This work offers critical information when designing surface modifiers and presents a simple and reproducible dual interface modification strategy to reproducibly achieve efficient and stable PSCs.

## Supplementary Material



## Notes

Figures S1–S16 show solar cell structure, distribution of the photovoltaic parameters, grain size distribution histograms, Kelvin Probe measurements, XPS high-resolution spectra of the perovskite films fabricated, UPS spectra of all films, Tauc plot calculation, energy level diagram, *J*–*V* curves, and extended discussion of TRPL of the perovskite films with different composition, box plots showing the distribution of the photovoltaic parameters for the solar cells, MPPT of the PSCs, Nyquist plot of the impedance, the circuit used to fit the impedance spectra, and the dielectric relaxation resistance *R*_ion_ and capacitance vs voltage *C*_ion_. (PDF)
