# Supplementary material for: Dual Interface Modification for Reduced Nonradiative Recombination in n–i–p Methylammonium-Free Perovskite Solar Cells
Source: ACS Appl Mater Interfaces. 2025 Jan 22;17(5):8610–8. doi: 10.1021/acsami.4c20462 (PMC12128035; doi:10.1021/acsami.4c20462)
Supplement: Supplementary file 1 [file am4c20462_si_001.pdf]

Supporting Information

Dual Interface Modification for Reduced Non-Radiative Recombination in n-i-p methylammonium-free Perovskite Solar Cells

Juan José Rodriguez-Perez[1,2], Diego Esparza[3], Muhammad Ans,[4] David Armando Contreras-Solorio[1], Teresa Diaz Perez[2], Jhonatan Rodriguez-Pereira[5,6], Eva M. Barea[2], Isaac Zarazua[7], Daniel Prochowicz,[4] Seckin Akin,[8] Juan P. Martinez-Pastor[9], Jorge Pascual[10], Iván Mora-Seró[2]* and Silver-Hamill Turren-Cruz[2,4,9]**

[1] Unidad Académica de Ciencia y Tecnología de la Luz y la Materia, Universidad Autónoma de Zacatecas, Carr. Zacatecas-Guadalajara km 6, Ejido La Escondida, 98160, Zacatecas, Zac., México.

[2]Institute of Advanced Materials (INAM), Universitat Jaume I, Av. Vicent Sos Baynat, s/n, 12071, Castellón de la Plana, Spain.

[3]Unidad Académica de Ingeniería Eléctrica, Universidad Autónoma de Zacatecas, Av. Ramón López Velarde 801, Col. Centro, 98060, Zacatecas, Zac., México.

[4]Department of Physical Chemistry, Polish Academy of Sciences, Warsaw 01-224, Poland.

[5]Center of Materials and Nanotechnologies, Faculty of Chemical Technology, University of Pardubice, Nam. Cs. Legii 565, 53002 Pardubice, Czech Republic.

[6]Central European Institute of Technology, Brno University of Technology, Purkyňova 123, 612 00 Brno, Czech Republic.

[7]Universidad de Guadalajara, Centro Universitario de los Lagos, Lagos de Moreno, Jal. 47460, México.

[8]Department of Metallurgical and Materials Engineering, Necmettin Erbakan University, 42060 Konya, Turkey.

[9]Instituto de Ciencia de los Materiales (ICMUV), Universitat de Valencia, 46980 Paterna, Spain

[10]Polymat, University of the Basque Country UPV/EHU, 20018 Donostia-San Sebastian, Spain.

*Corresponding author: jorge.pascual@polymat.eu, sero@uji.es, silver.turren@uv.es

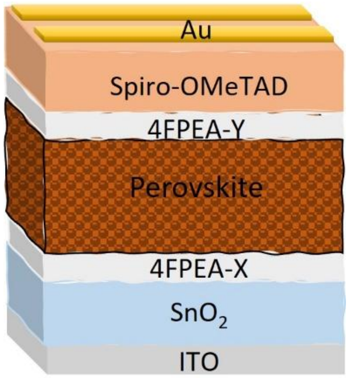

Figure S1. Schematic of the perovskite solar cell structure containing the interfacial modification.

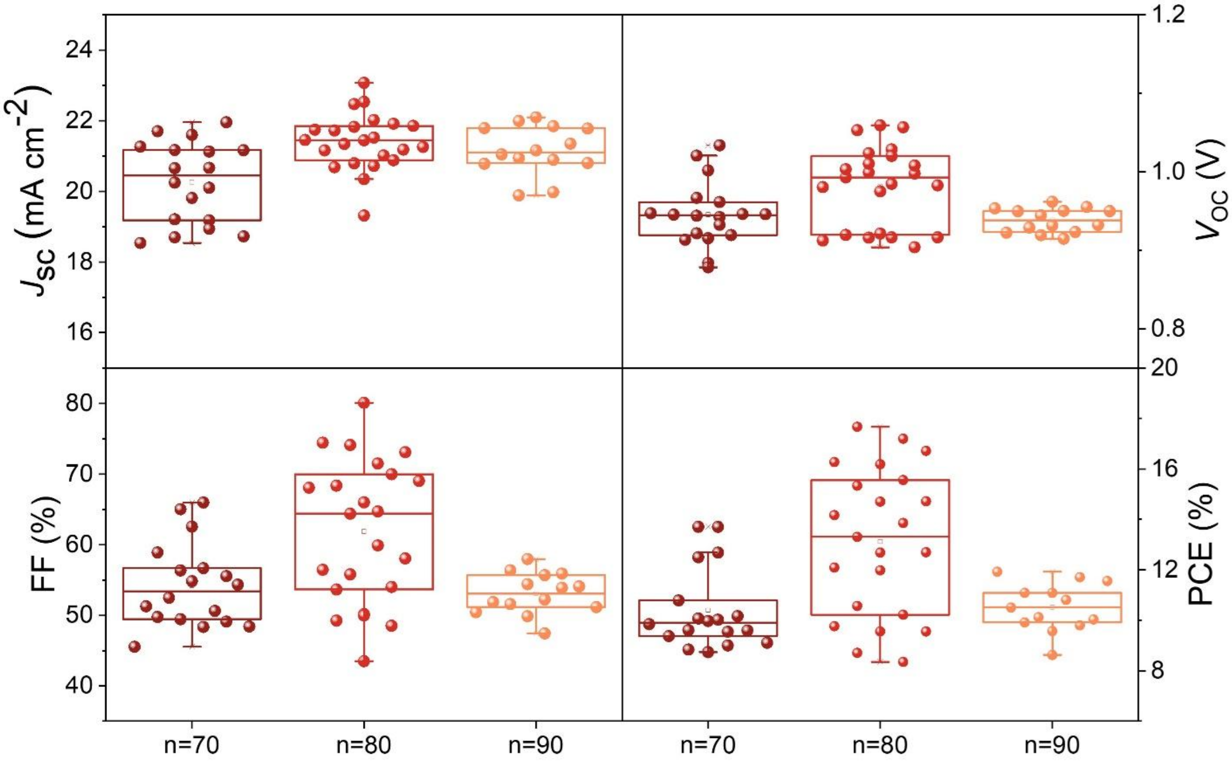

Figure S2. Distribution of the photovoltaic parameters extracted from the *J*-*V* curves for PSCs fabricated with different DipI concentrations for nominal *n* values of 70, 80 and 90. The variable *n* denotes the dimensions of the two-dimensional slabs.

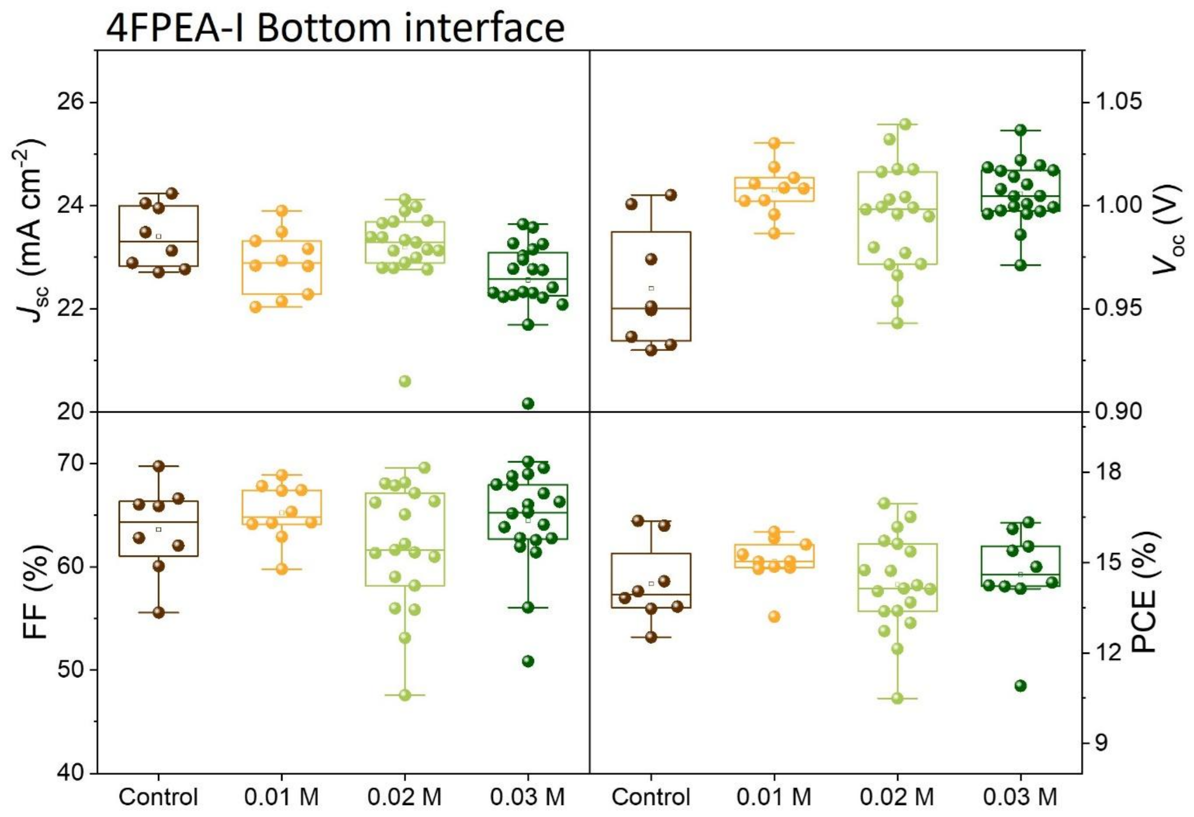

Figure S3. Distribution of the photovoltaic parameters extracted from the *J*-*V* curves for PSCs fabricated with different 4FPEA-l concentrations for perovskite bottom interface modification.

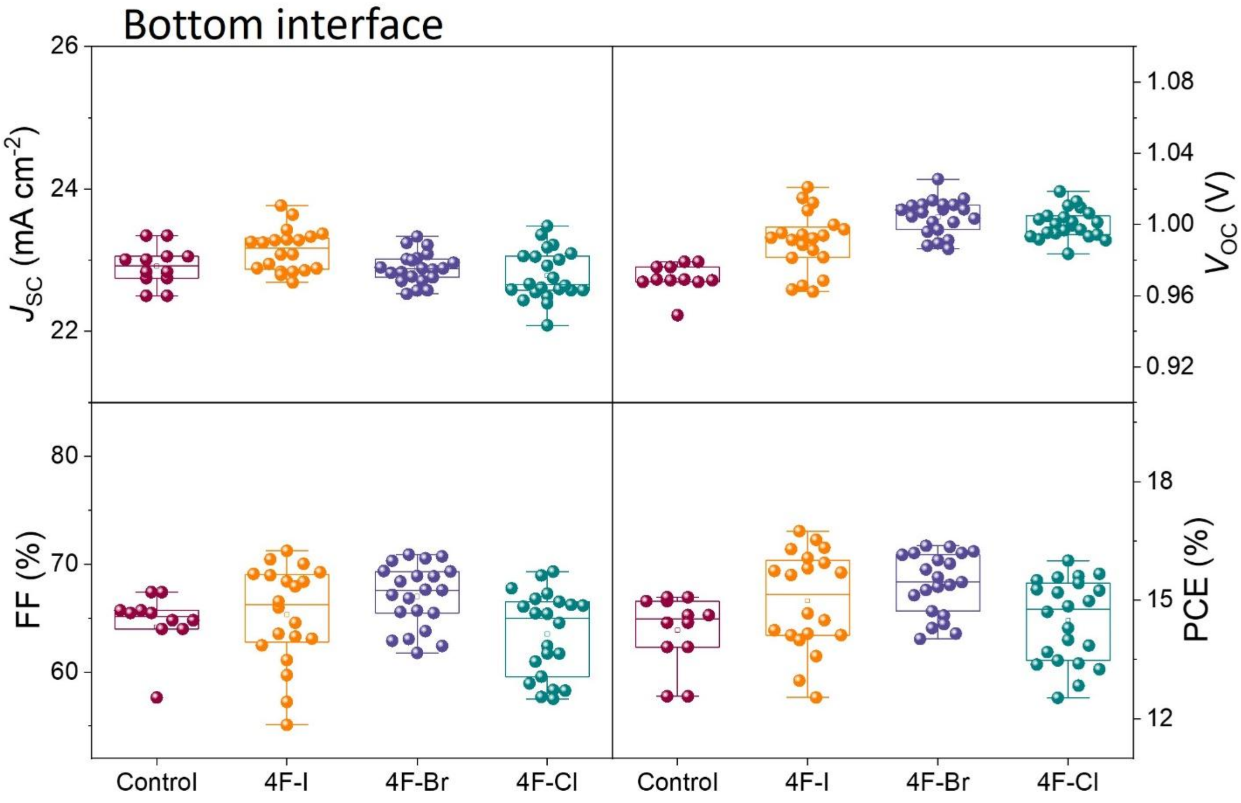

Figure S4. Distribution of the photovoltaic parameters extracted from the *J*-*V* curves for PSCs fabricated with different interlayer modifications on the bottom interface.

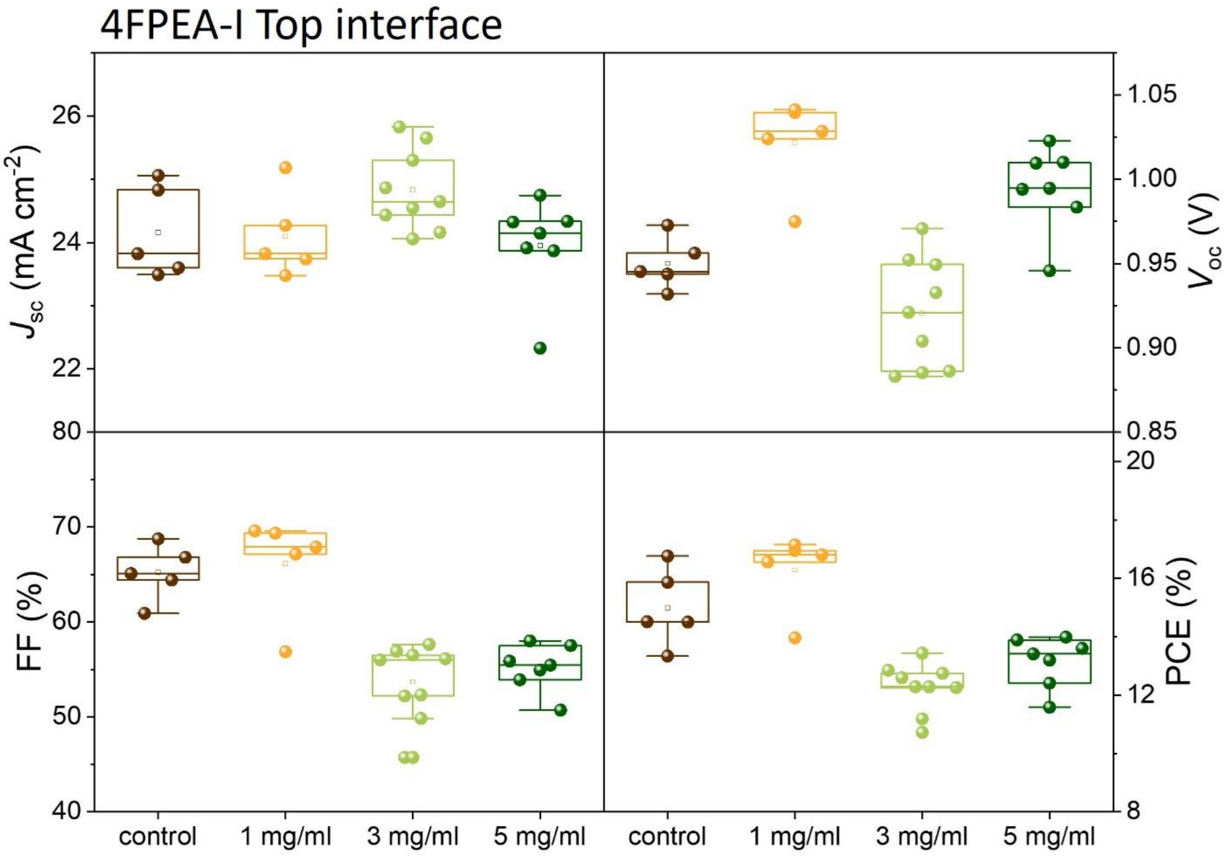

Figure S5. Distribution of the photovoltaic parameters extracted from the *J*-*V* curves for PSCs fabricated with different 4FPEA-I concentrations for perovskite top interface modification.

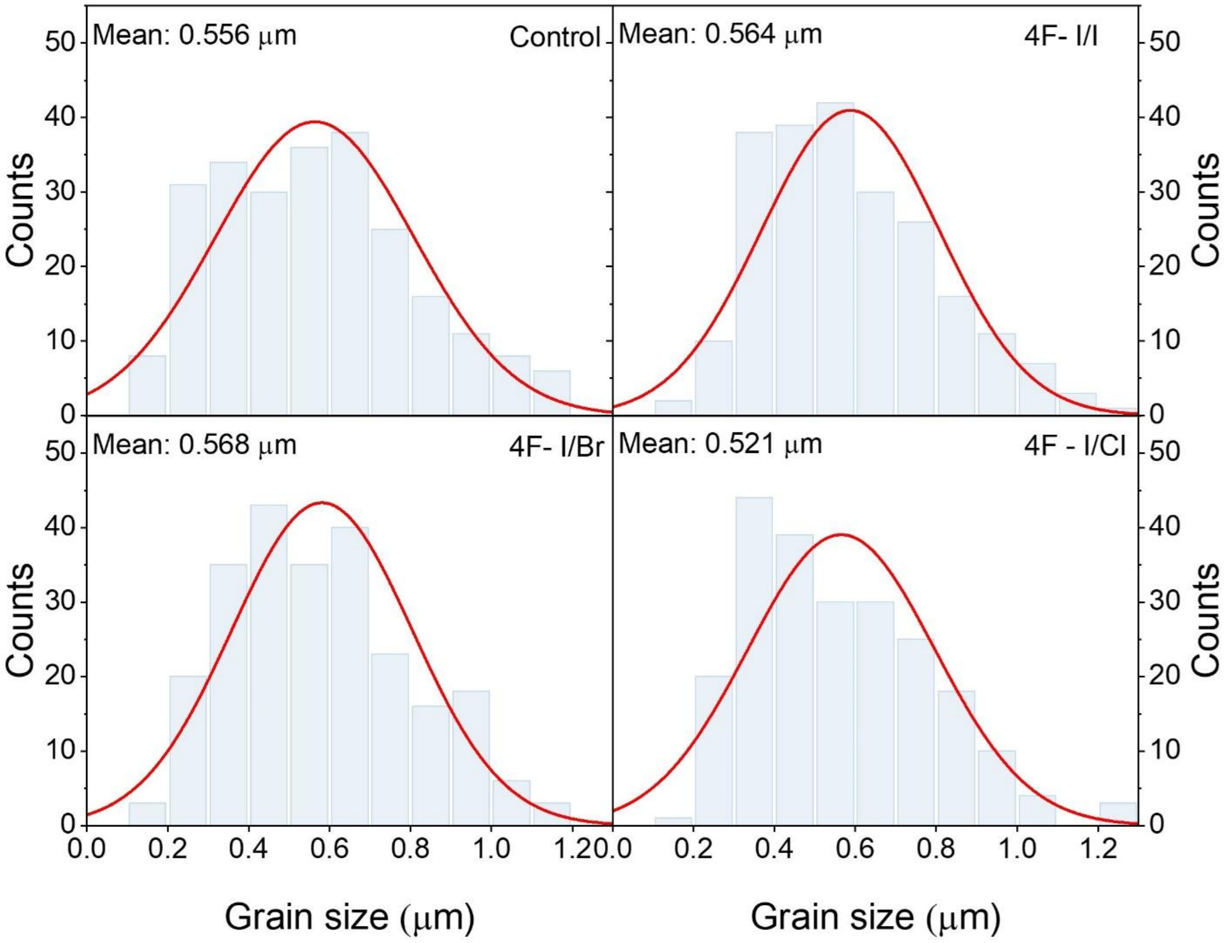

Figure S6. Grain size distribution histograms of control and interface-modified perovskite thin films.

Table S1. Data from the grain size distribution histograms of the control and interface-modified perovskite thin films from **Figure S6**.

Sample	Minimum (μm)	Median (μm)	Maximum (μm)
**Control**	0.107	0.56 ± 0.25	1.18
**4F-I/Br**	0.142	0.57 ± 0.22	1.16
**4F-I/I**	0.160	0.56 ± 0.22	1.31
**4F-I/Cl**	0.195	0.52 ± 0.23	1.34

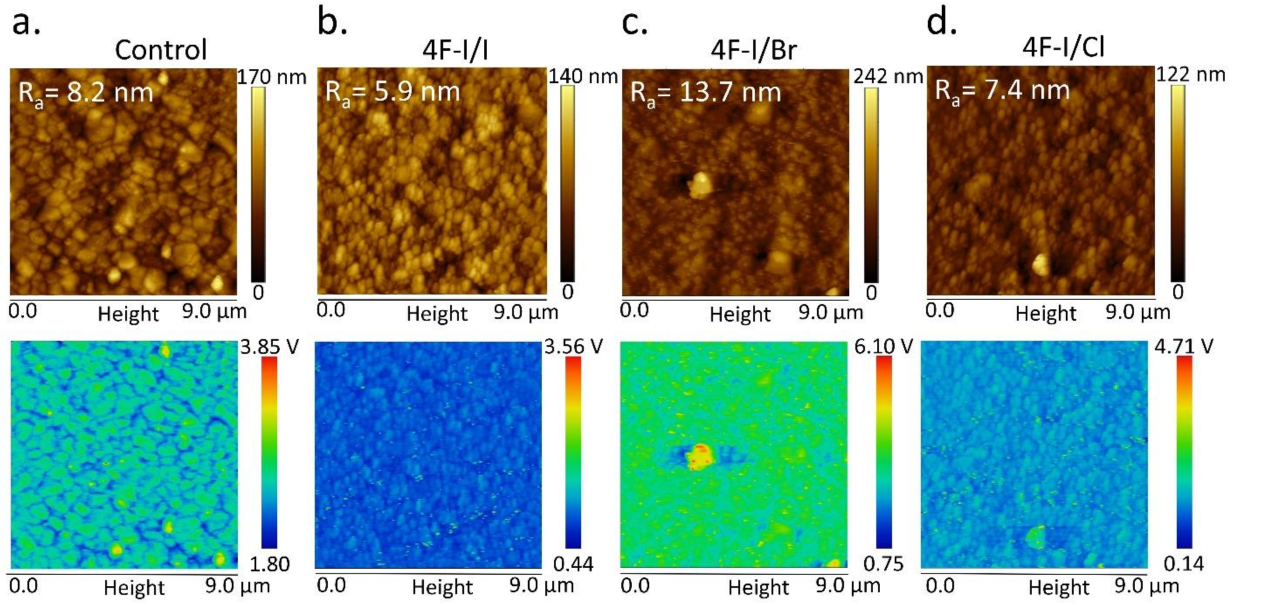

Figure S7. Kelvin Probe measurements for a) control and with interface modifications b) 4F-I/I, c) 4F-I/Br and d) 4F-I/Cl.

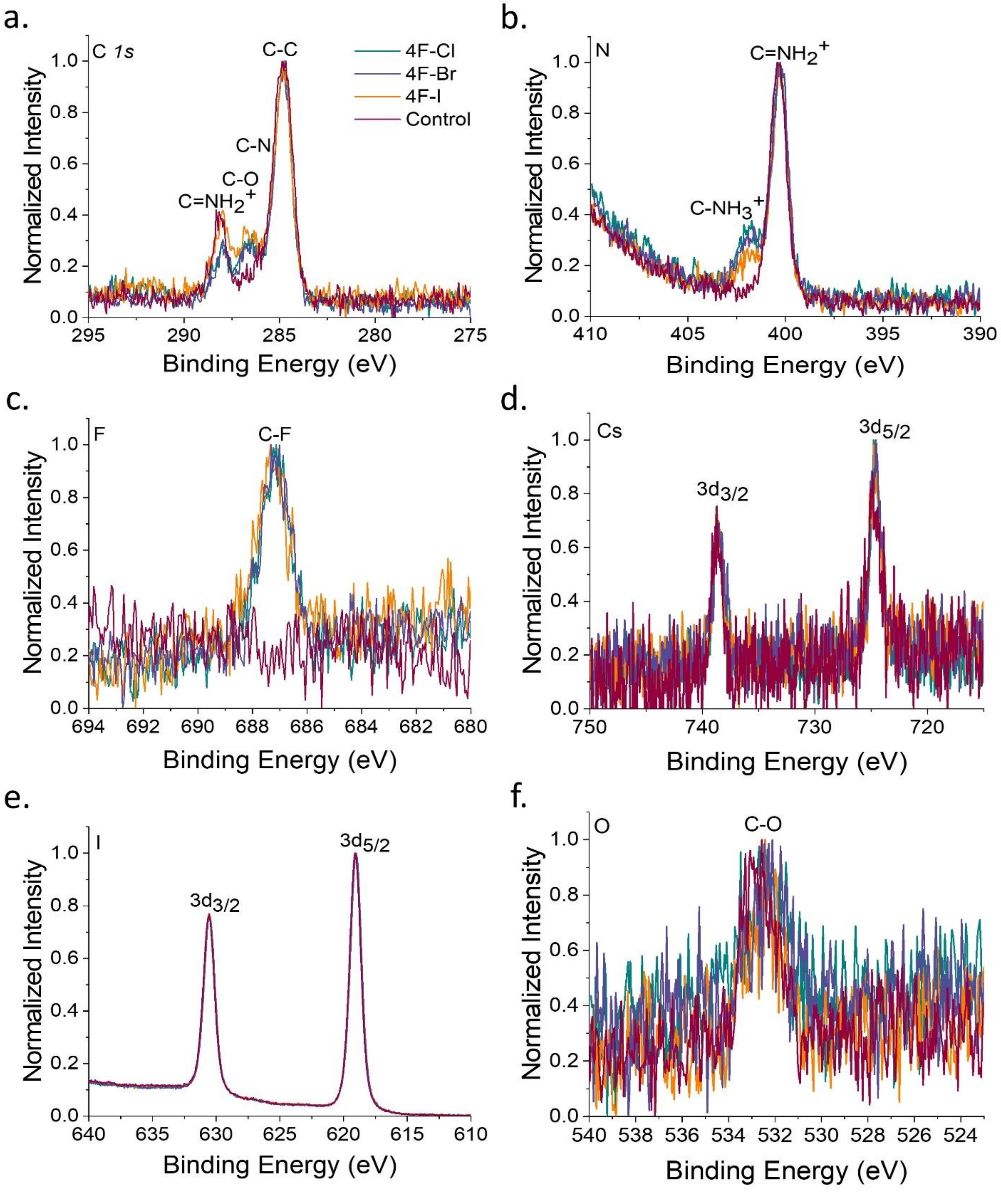

Figure S8. XPS high resolution spectra of: a) C 1*s*, b) N 1*s*, c) F 1*s*, d) Cs 3*d*, e) I 3*d* and f) O 1*s* peaks.

Table S2. Surface composition of the control and different interlayer modification films.

Sample Identifier	Atomic concentration (%)												
	**C**-(C,H)	**C**-N	**C**-O, **C**-F	**C**=NH_2^+	C-**O**	**I**$^-$	**Pb**$^{2+}$	**Cs**$^+$	C-**N**H_2^+	C-**N**H_3^+ **N**H_4^+	C-**F**	**Br**$^-$	**Cl**$^-$
**Control**	25.56	2.63	2.15	8.21	3.02	33.75	10.51	0.33	13.83	-	-	-	-
**4F-I/I**	20.81	3.23	5.43	7.15	1.69	34.62	9.65	0.37	12.13	1.82	3.12	-	-
**4F-I/Br**	25.90	2.74	5.73	5.51	1.57	30.77	8.63	0.42	10.55	2.70	4.56	0.92	-
**4F-I/Cl**	26.00	2.43	6.01	5.25	1.74	31.70	9.00	0.52	9.96	2.78	3.76	-	0.85

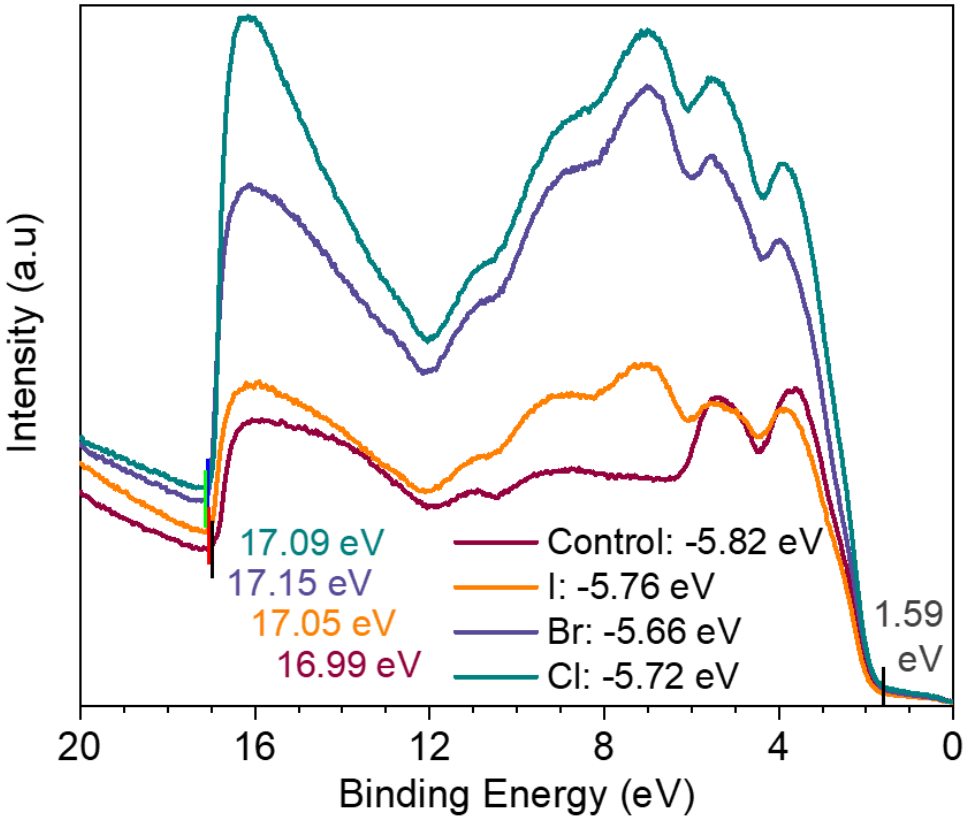

Figure S9. UPS spectra of all films. The linear interpolations reveal the photoemission cut-off energy boundary (E_{cutoff}) along with the UPS photoemission onset energy (E_{onset}), respectively. The work function of the film can be derived by subtracting the E_{cutoff} value from 21.22 eV.

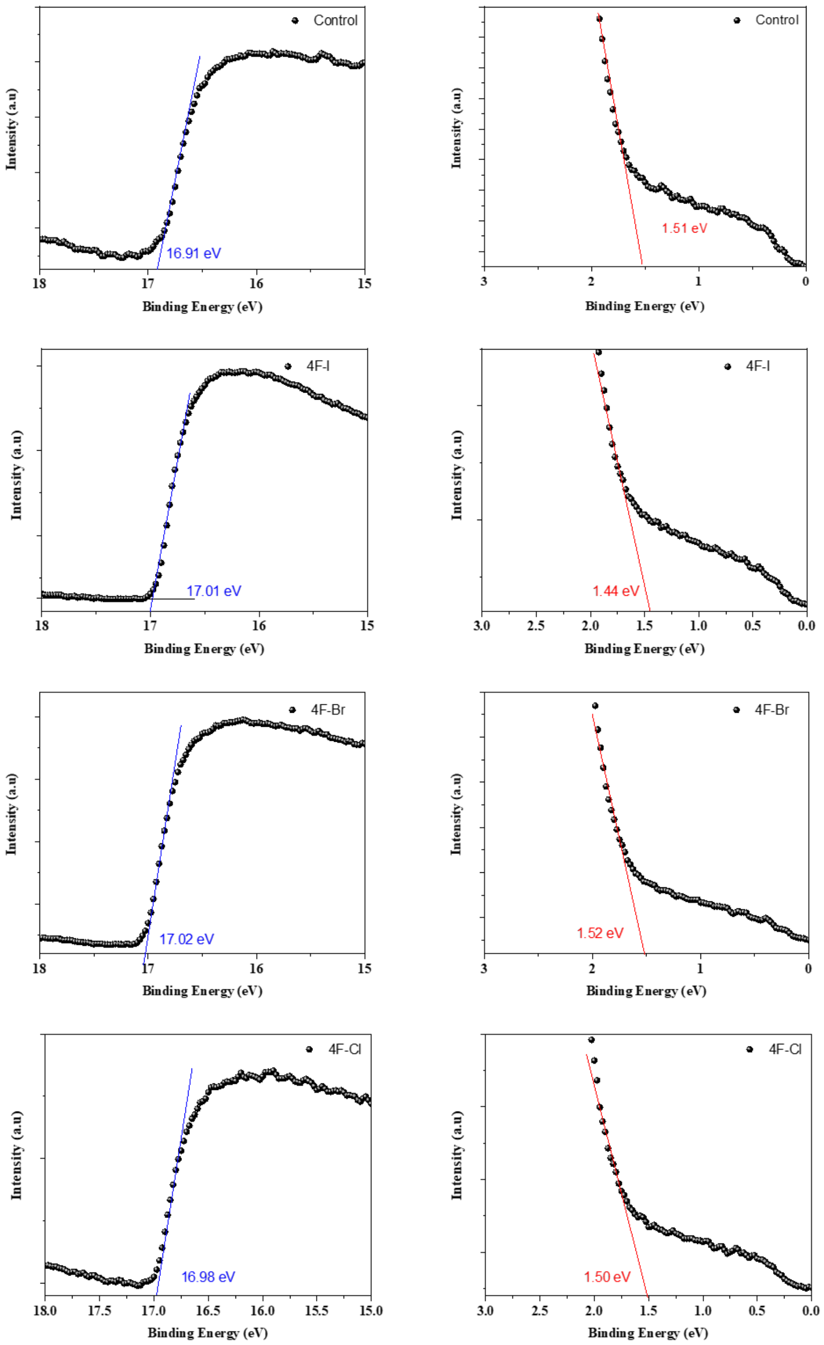

Figure S10. UPS spectra for each film.

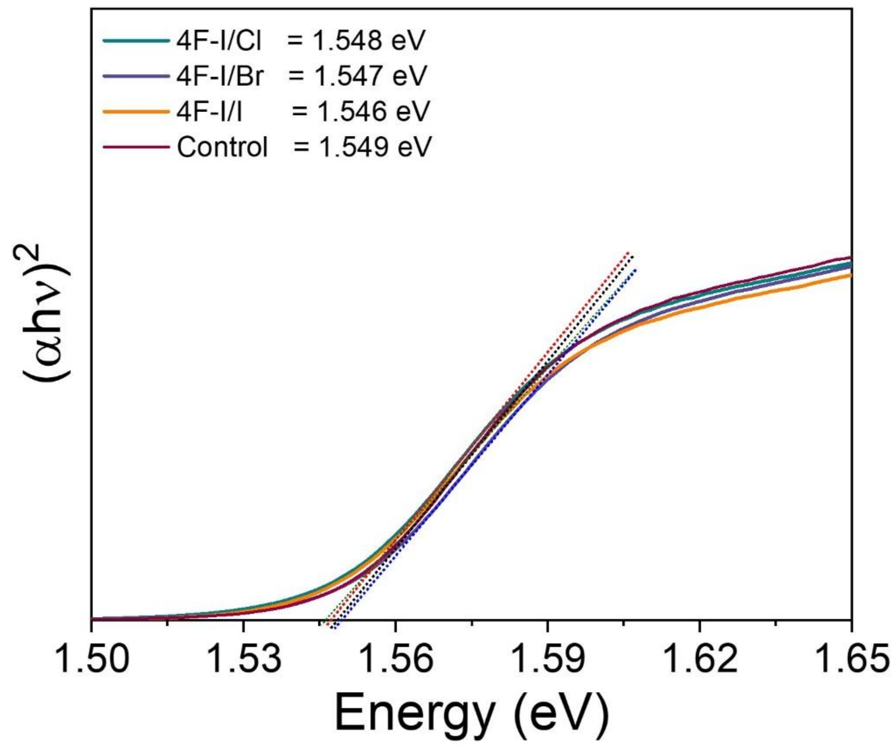

Figure S11. Tauc plot of control PSCs and with interfacial modifications.

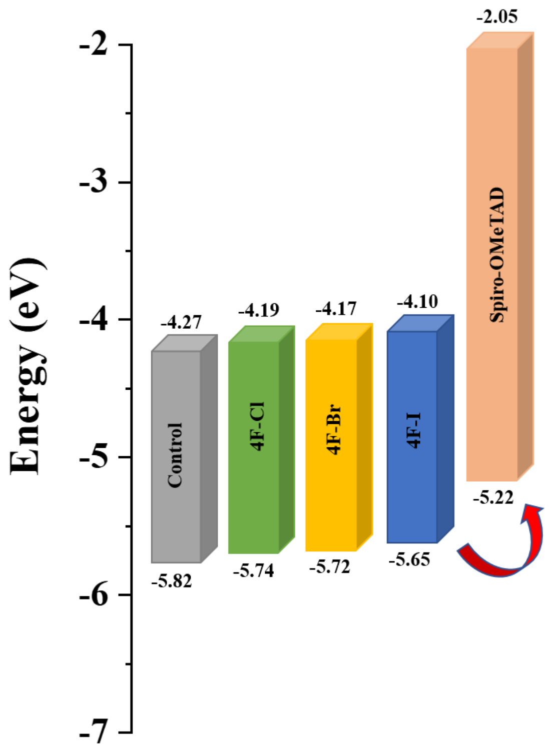

Figure S12. Energy level diagram for control perovskite films and treated with the different interface modifiers.

Table S3. Decay lifetime parameters of the control and interface-modified samples.

Sample	λ_{em} (nm)	τ_f (ns)	τ_s (ns)	τ_{ef} (ns)
**Control**	803	591.82	597.75	594.80
**4F-I/I**	803	762.03	777.37	769.78
**4F-I/Br**	803	441.85	446.58	444.22
**4F-I/Cl**	803	838.22	853.76	846.06

The TRPL measurements are shown in **Figure 2c**; for the control and 4F-I/Br samples, the excitation bandwidth is 3 nm, and emission is 6 nm, with an intensity of 100% with an integration time of 1 s and a wavelength step of 1 nm, for the samples 4F-I/Cl and 4F-I/I they were carried out with an intensity of 300%.

Analyzing decay curves, the relaxation dynamics can be a bipotential function $I(t) = A_1e^{(-t/\tau f)} + A_2e^{(-t/\tau s)}$, where I is related to the time-dependent PL current (t), A_1, τ_f y A_2, τ_s are the fast and slow decay time parameters, respectively, to calculate the effective lifetime through $\tau_{ef} = (\Sigma A_i\tau_i^2/\Sigma A_i\tau_i)$.

Table S4. Average (in brackets) and champion photovoltaic parameter values obtained from the *J*–*V* scans of the control and interface-modified PSCs.

Sample	J_{SC} (mA cm^{-2})	V_{OC} (V)	FF (%)	PCE (%)
**Control**	**22.8** (22.0±0.4)	**1.07** (1.05±0.01)	**75.0** (72.3±1.7)	**17.8** (16.5±0.6)
**4F-I/I**	**22.9** (22.2±0.4)	**1.12** (1.10±0.01)	**78.6** (76.6±1.3)	**20.0** (18.7±0.6)
**4F-I/Br**	**22.8** (22.0±0.4)	**1.11** (1.07±0.01)	**77.4** (74.7±1.5)	**19.3** (17.8±0.6)
**4F-I/Cl**	**22.9** (22.2±0.4)	**1.10** (1.09±0.01)	**78.3** (76.4±1.2)	**19.3** (18.3±0.5)

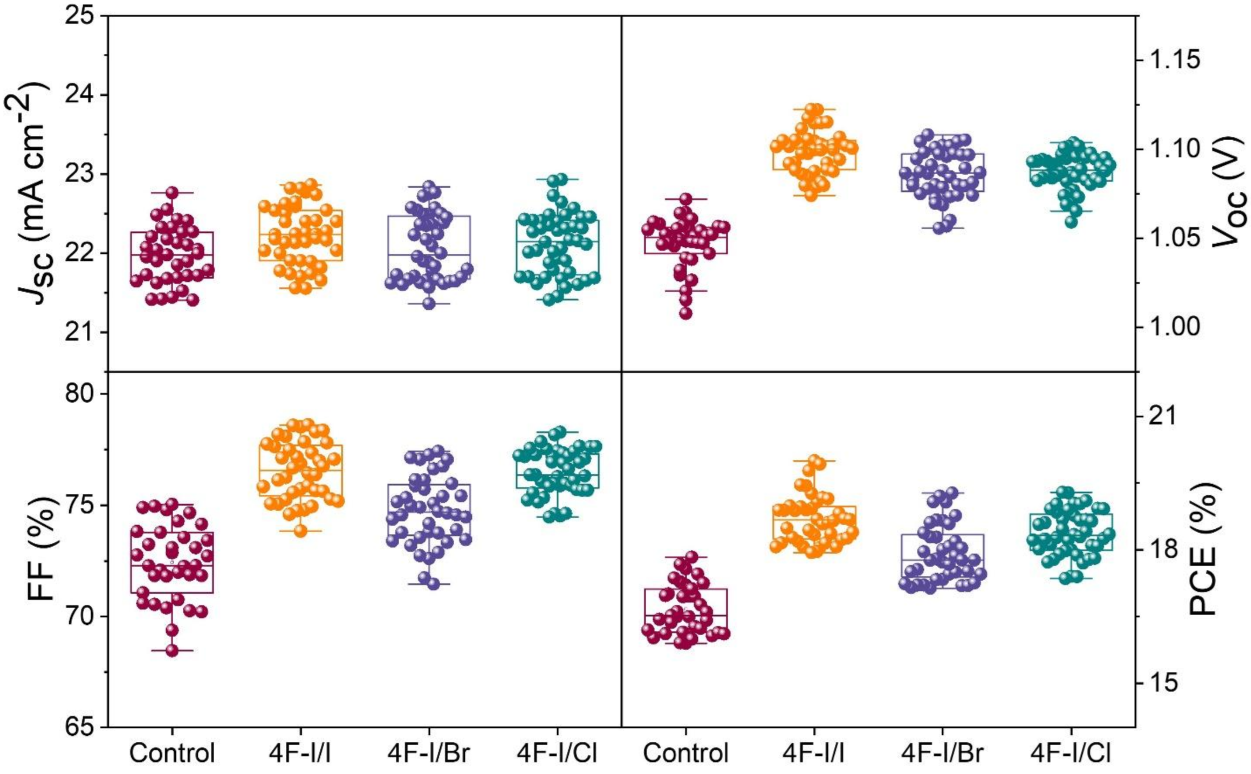

Figure S13. Distribution of the photovoltaic parameters for the control and interface-modified PSCs.

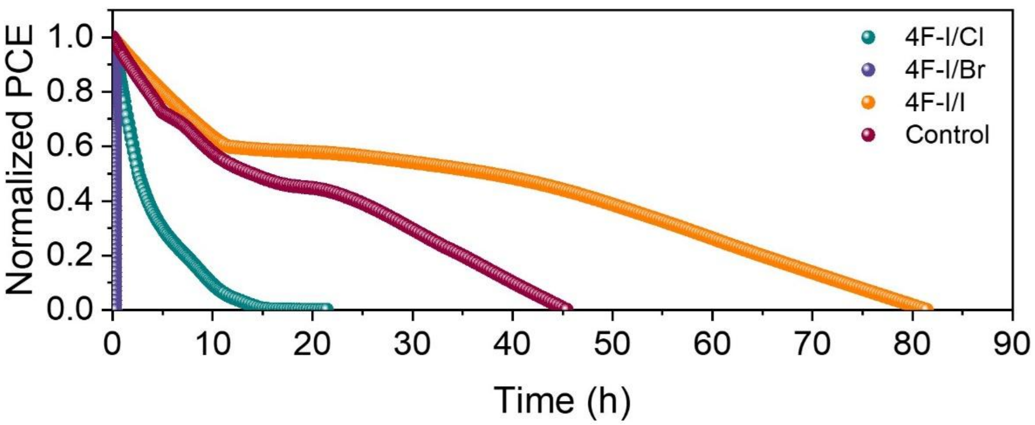

Figure S14. MPPT of control and interface-modified PSCs. The measurement was carried out without encapsulation in the ambient atmosphere at 70% RH without temperature control (temperature reaching 60°C due to lamp heat radiation),

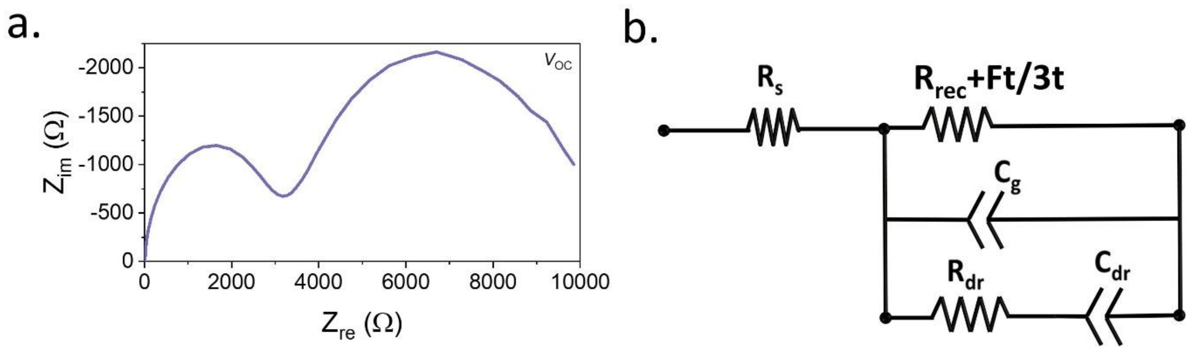

Figure S15. a) Nyquist plot of the impedances for the sample control measured under open-circuit conditions at several irradiances and b) circuit used to fit the impedance spectra, for better fitting constant phase elements have been used instead of ideal capacitors.

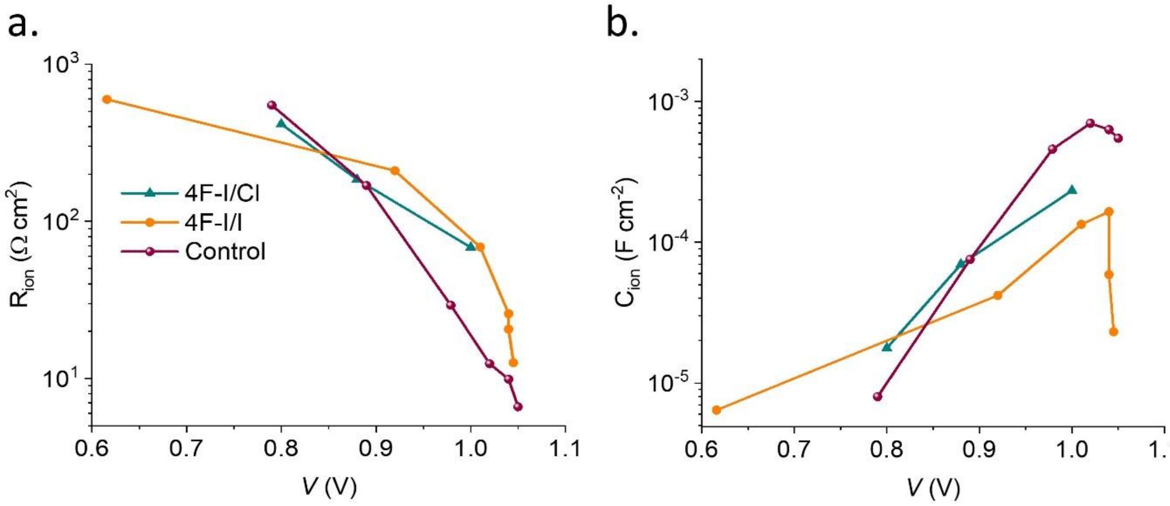

Figure S16. a) Dielectric relaxation resistance and b) capacitance vs voltage of control and interface-modified PSCs.
